# Notes and News

**Published:** 1902-06-14

**Authors:** 


### Notes and News.

With this number of "The Hospital" is issued a Special Coronation Hospital Sunday Appeal Supplement and other supplements in the form of excellent photogravure portraits of the Prince and Princess of Wales.

By special permission of the Prince and Princess of Wales the frontispiece of the Hospital for Sick Children, Great Ormond Street, Coronation Bazaar souvenir will consist of a photograph of Prince Edward by Mr. Richard N. Speaight. The souvenir will be unique of its kind, as it will consist entirely of photographs of the children of the patrons and stallholders.

Among the appointments at University College which have recently been made there appear the names of Dr. Dudley Buxton, who has been re-appointed senior anæsthetist to the hospital; Dr. Charles Bolton, reappointed resident medical officer; and Mr. W. B. L. Trotter, F.R.C.S., reappointed surgical registrar. Mr. C. F. Hardie has been awarded the Atchison scholarship for having shown the best work and the greatest proficiency as a student of the medical faculty during the preceding two years. Dr. Angel Money has been appointed to represent the college at the jubilee of the University of Sydney.

Unless the trustees of the Manchester Royal Infirmary should intervene at the last moment, which seems hardly likely, it appears as if, after all the discussions on the subject, the infirmary will be rebuilt on the old site. The plans were passed some time ago subject to certain alterations and will be ready for final presentation in July, and it is expected that the trustees will then be guided by the board. That all people will not be satisfied goes without saying. The site covers just over four acres, a good deal of which will have to be preserved unbuilt upon. On this site it is proposed to erect a hospital for 457 patients, so that the building, when it is completed, will have to provide accommodation for not far short of 700 persons. Although there is a strong feeling in Manchester in favour of keeping the old infirmary on the old site there are plenty of people who object, maintaining that the place, surrounded as it is on every side by busy streets and situated in the very centre of the fog and grime of the city, is not suited for a hospital.

The muzzling order has had to be re-imposed in parts of Devonshire and Cornwall.

We are informed by the Director-General, Army Medical Service, that the Secretary of State for India has decided to approve an increase to the pay of officers of the Royal Army Medical Corps below the rank of major while serving in India, and also of the issue of charge allowances for senior medical officers of station hospitals there. We are extremely glad to be able to make this announcement because there can be no doubt that what has been called the Indian grievance has long been regarded as a great hardship, if not an actual breach of faith.

At the meeting of the Metropolitan Public Gardens Association held last week, Lord Meath being in the chair, considerable discussion arose in reference to faculties which had recently been granted by the Consistory Court, for the erection of buildings on churchyards, some of which had even been laid out as public gardens, and a strong feeling was expressed that if the Disused Burial Grounds Act should not prove strong enough to prevent this happening, it should be strengthened without delay. No one can doubt the enormous benefit which accrues to many poor and crowded districts from the operations of the Association, by whose exertions so many small and well protected spots have been rendered available as airing spaces for the children of the poor.

The festival dinner of the London Medical Graduates' College and Polyclinic took place last week at the Criterion Restaurant, Mr. Asquith, K.C., M.P., presiding. In proposing the toast of "The London Polyclinic," Mr. Asquith said that this was a charitable institution in the best sense of the word. Without coming in the least degree into competition or rivalry with the hospitals, it provided a means by which persons who could not afford themselves to pay the usual fee for consultation, could be brought by their doctor to the polyclinic and there get in consultation with that doctor, the best medical advice that the most skilled members of the profession in London could supply. That supplied a gap in our philanthropic machinery, and he thought it could not be too highly valued.

While Liverpool is anxious to have a University all to itself, Manchester seems, from the account of a meeting held last week, to be desirous of laying hands upon the title "Victoria University" and changing it into the "Victoria University of Manchester." This seems rather sharp practice, and we can hardly wonder that Leeds objects to these proposals and considers that the time has come when the Yorkshire College is bound to take up a firm attitude of opposition to the disruption of the Victoria University. At a meeting held on June 5th at the Leeds Philosophical Hall, it was resolved to present a petition to his Majesty in Council in opposition to the petitions of University College, Liverpool, and of Owens College, Manchester, for charters as separate Universities, and to any other scheme tending to the dismembership of the Victoria University.

